# Effect of duodenal-jejunal bypass on diabetes in the early postoperative period

**DOI:** 10.1038/s41598-023-28923-3

**Published:** 2023-02-01

**Authors:** Shohei Okikawa, Hideya Kashihara, Mitsuo Shimada, Kozo Yoshikawa, Takuya Tokunaga, Masaaki Nishi, Chie Takasu, Yuma Wada, Toshiaki Yoshimoto

**Affiliations:** Department of Surgery, Tokushima University, 3-18-15 Kuramoto-Cho, Tokushima, Tokushima 770-8503 Japan

**Keywords:** Endocrine system and metabolic diseases, Gastroenterology

## Abstract

Metabolic surgery is an effective treatment for patients with type 2 diabetes mellitus (T2DM). The aim of this study was to investigate the effect of duodenal-jejunal bypass (DJB) in a rat model of T2DM during the early postoperative period. A rat model of non-obese T2DM was allocated to two groups: a sham group and a DJB group. On postoperative day 1 (1POD), oral glucose tolerance testing (OGTT) was performed and the changes of glucose transporter expressions in the small intestine was evaluated. [18F]-fluorodeoxyglucose ([18]-FDG) uptake was measured in sham- and DJB-operated rats using positron emission tomography-computed tomography (PET-CT). DJB improved the glucose tolerance of the rats on 1POD. The expression of sodium-glucose cotransporter 1 (SGLT1) and glucose transporter 1 (GLUT1) was high, and that of GLUT2 was low in the alimentary limb (AL) of rats in the DJB group. PET-CT showed that [18F]-FDG uptake was high in the proximal jejunum of DJB-operated rats. These results may show that DJB improve glucose tolerance in very early postoperative period as the result of glucose accumulation in the AL because of changes in glucose transporter expression.

## Introduction

Type 2 diabetes mellitus (T2DM), which is underpinned by both insulin resistance and a deficiency in insulin secretion^1^, is the most common type of diabetes, accounting for 90–95% of all cases^2^. It is predicted that the number of patients with T2DM will continue to increase over the next 20 years^3^, with the number of patients reaching 700 million by 2045^4^. Thus, T2DM has become an important global public health issue.

Recently, the surgical treatment of T2DM has become common^5–7^. Duodenal-jejunal bypass (DJB), a procedure that involves the anastomosis of the distal jejunum to the duodenum and the diversion of biliopancreatic juice to the ileum, is a type of metabolic surgery that ameliorates hyperglycemia without changing the anatomy of the stomach or requiring dietary restriction^8,9^. Our previous study showed that DJB ameliorates insulin resistance by increasing glucagon-like peptide-1 secretion, secondary to an increase in bile acid concentration, 8 weeks after the surgery^10^. Interestingly, Seki et al*.* reported that a combination of sleeve gastrectomy (SG) and DJB in patients with T2DM and poor medical control substantially reduces the required dose of insulin on postoperative day 1 (1POD), and on or after day 2 (2POD) no antidiabetic medication was required^11^. This suggests that metabolic surgery, including DJB, rapidly ameliorates hyperglycemia in the very early postoperative period. However, the mechanism of this rapid improvement in T2DM remains to be determined.

Recently, it has become clear that the mechanisms whereby T2DM is improved differs according to the surgical method used. Several studies have shown that intestinal glucose uptake secondary to remodeling of the intestine is important for the effects of Roux-en-Y gastric bypass (RYGB) on T2D^12,13^. It has been reported that RYGB induces morphological adaptation, including mucosal hyperplasia and hypertrophy, in the alimentary limb (AL)^12–14^. This remodeling of the intestinal mucosa induces a reprogramming of glucose metabolism and increases metabolic rate to meet the higher energy demand, which causes an increase in carbohydrate consumption by the gut^12,13,15^.

Monosaccharides, including d-glucose, which is crucial for energy production, cannot readily cross cell membranes because they are hydrophilic. Therefore, transporters expressed in the luminal brush border membrane (BBM) and basolateral membrane (BLM) of small intestinal epithelial cells are necessary for the absorption of monosaccharides^16,17^. The absorption of d-glucose in the small intestine is principally mediated by sodium-glucose cotransporter 1 (SGLT1) and glucose transporter 2 (GLUT2)^18^. SGLT1, which is constitutively expressed in the BBM of enterocytes, transports glucose from the intestinal lumen into the epithelial cells, and GLUT2, which is expressed in the BLM, transports glucose from epithelial cells into the vascular lumen^19^. GLUT1, which is responsible for the basolateral uptake of glucose, is usually expressed at very low levels in the mature intestine^20^. However, it has been reported that GLUT1 expression is upregulated in the BLM of epithelial cells of the AL after RYGB^12,13^, and the resulting increase in glucose supply to small intestinal epithelial cells is required for the intestinal remodeling that occurs following surgery^12^. These changes in the intestinal mucosa are caused by exposure to undigested food in the AL. In contrast, SG, a metabolic surgery that does not cause the diversion of nutrient flow, does not cause intestinal remodeling^12–14^. RYGB and DJB are similar techniques, in that they alter nutrient flow through the gut, but there have been no studies to date that have investigated whether these changes occur during the early postoperative period. Therefore, in the present study, we aimed to determine the mechanism whereby T2DM is improved by DJB during the very early postoperative period, focusing on changes in the expression of glucose transporters in the small intestine.

## Results

### DJB rapidly ameliorates hyperglycemia in rats with T2DM

**Figure 1:**
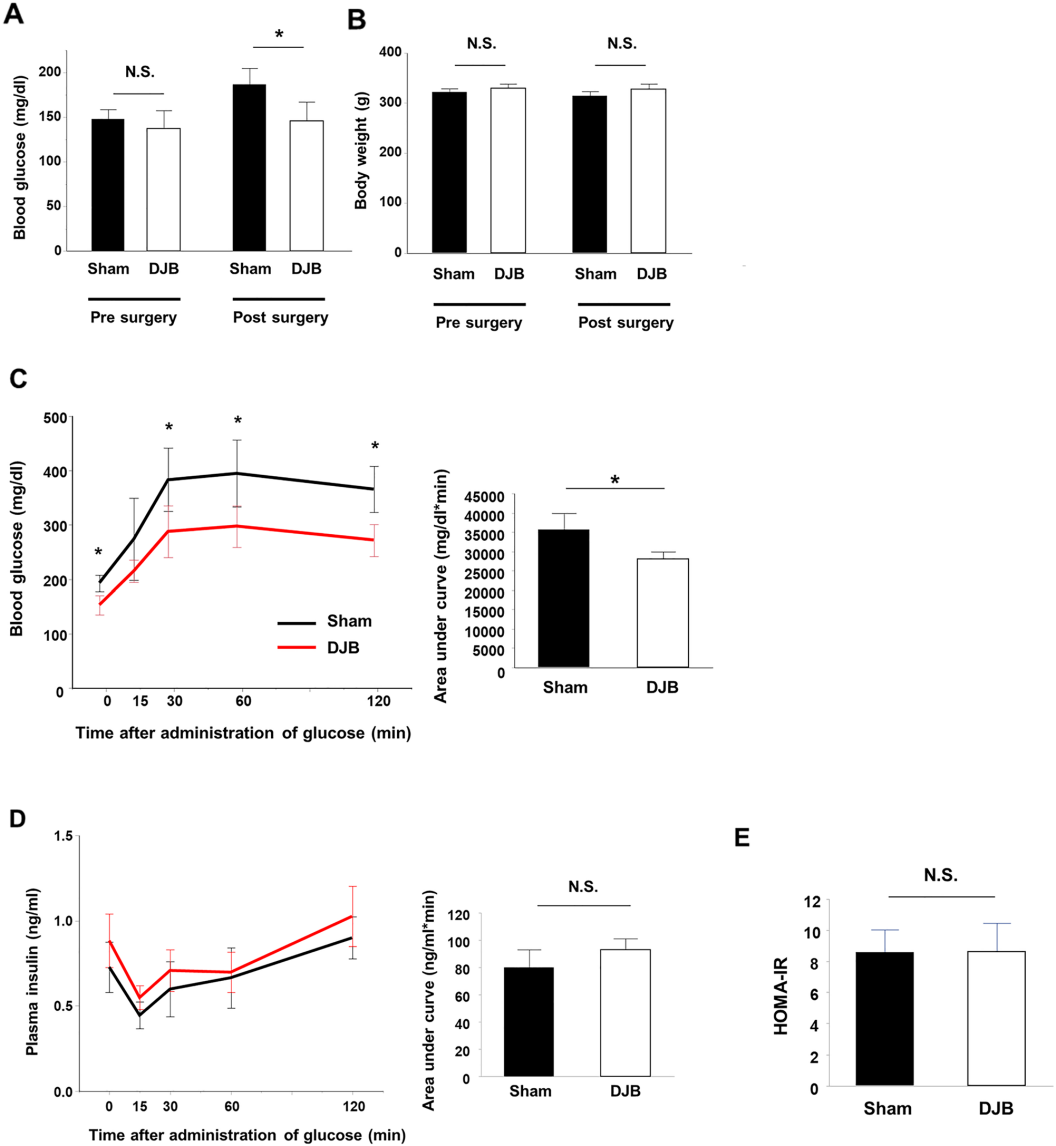
Duodenal-jejunal bypass (DJB) ameliorates hyperglycemia and improves glucose tolerance on postoperative day 1 (1POD). (**A**) Fasting blood glucose concentrations pre- and postoperatively in the sham and DJB groups. (**B**) Body mass during the preoperative and postoperative periods in the sham and DJB groups. (**C**) Changes in blood glucose concentration after glucose administration (2 g/kg) on 1POD in the sham and DJB groups. (**D**) Changes in plasma insulin concentrations after glucose administration (2 g/kg) on 1POD in the sham and DJB groups. (**E**) Homeostasis model of assessment-insulin resistance (HOMA-IR) on 1POD in the sham and DJB groups. Data are presented as mean ± SD. *P < 0.05; *n.s.* not significant (Student’s *t*-test).

The preoperative fasting blood glucose concentrations and body masses of the rats did not significantly differ between the groups (Fig. 1A,B). Although there were no differences in the body masses between the groups during the very early postoperative period, the fasting blood glucose concentrations of the DJB group were significantly lower than those of the sham group (*P* = 0.03) (Fig. 1A,B). OGTT revealed a rapid improvement in glucose tolerance in the DJB group, resulting in a markedly lower blood glucose concentration from 30 min after glucose administration in the DJB group than in the sham group (Fig. 1C). However, there were no significant differences in the plasma insulin concentrations during OGTT between the groups (Fig. 1D). HOMA-IR, a simple index of insulin resistance, did not differ between the two groups (Fig. 1E). These results suggest that DJB rapidly improves glucose tolerance, without ameliorating insulin resistance.

### Expression of glucose transporters in the small intestine is altered by DJB

**Figure 2:**
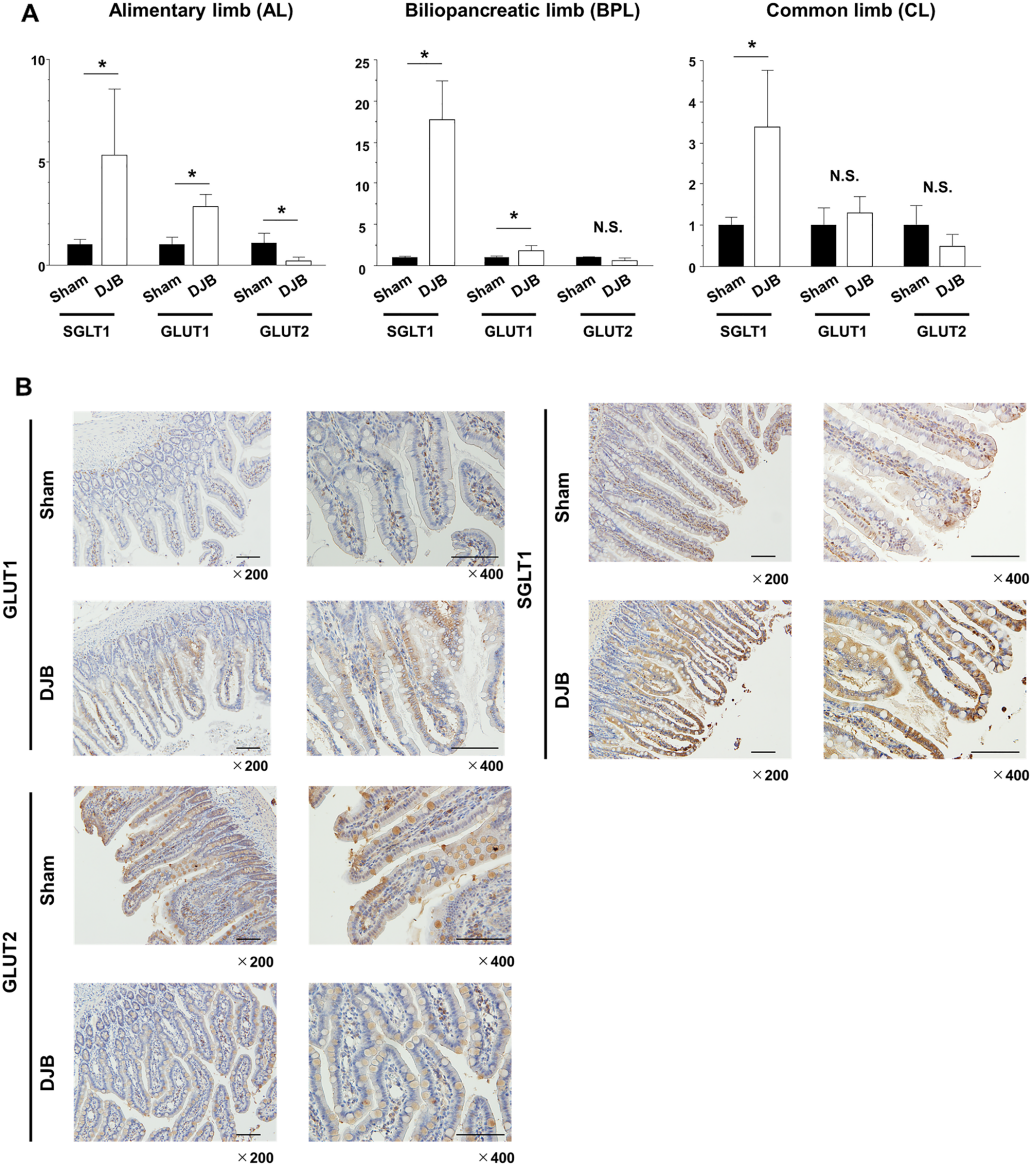
Glucose transporter expression changes rapidly after duodenal-jejunal bypass (DJB), especially in the alimentary limb. (**A**) Comparison of the glucose transporter mRNA expression after surgery in each section of the small intestine in the sham and DJB groups. (**B**) Immunohistochemistry to demonstrate the expression of glucose transporter 1, sodium-glucose cotransporter 1, and glucose transporter 2 of alimentary limb in the sham and DJB groups. Data are presented as mean ± SD. *P < 0.05; n.s., not significant (Student’s *t*-test).

To identify the cause of the reduction in hyperglycemia during very early postoperative period, we measured the mRNA expression of the glucose transporters SGLT1, GLUT1, and GLUT2 in the small intestine of the rats. Samples of small intestine were obtained from the AL, BPL, and CL of the DJB group and from the corresponding segments of the small intestine in the sham group. We found that SGLT1 mRNA expression was much higher in the AL, BPL, and CL of the DJB group. In addition, GLUT1 mRNA expression was significantly higher in the AL of the DJB group than in the equivalent parts of the intestine of the sham group (*P* = 0.02). In contrast, GLUT2 mRNA expression was significantly lower in the AL of the DJB group than in the equivalent part of the intestine in the sham group (*P* = 0.03) (Fig. 2A). We obtained similar results at the protein level by immunohistochemical staining of the intestines: SGLT1 and GLUT1 staining was more intense in the AL of the DJB group than in the equivalent part of the intestine of the sham group (Fig. 2B). These rapid changes in glucose transporter expression might contribute to the improvement in glucose tolerance in the very early postoperative period.

### [18F]-FDG PET-CT reveals that glucose is trapped in the small intestine of rats that undergo DJB

**Figure 3:**
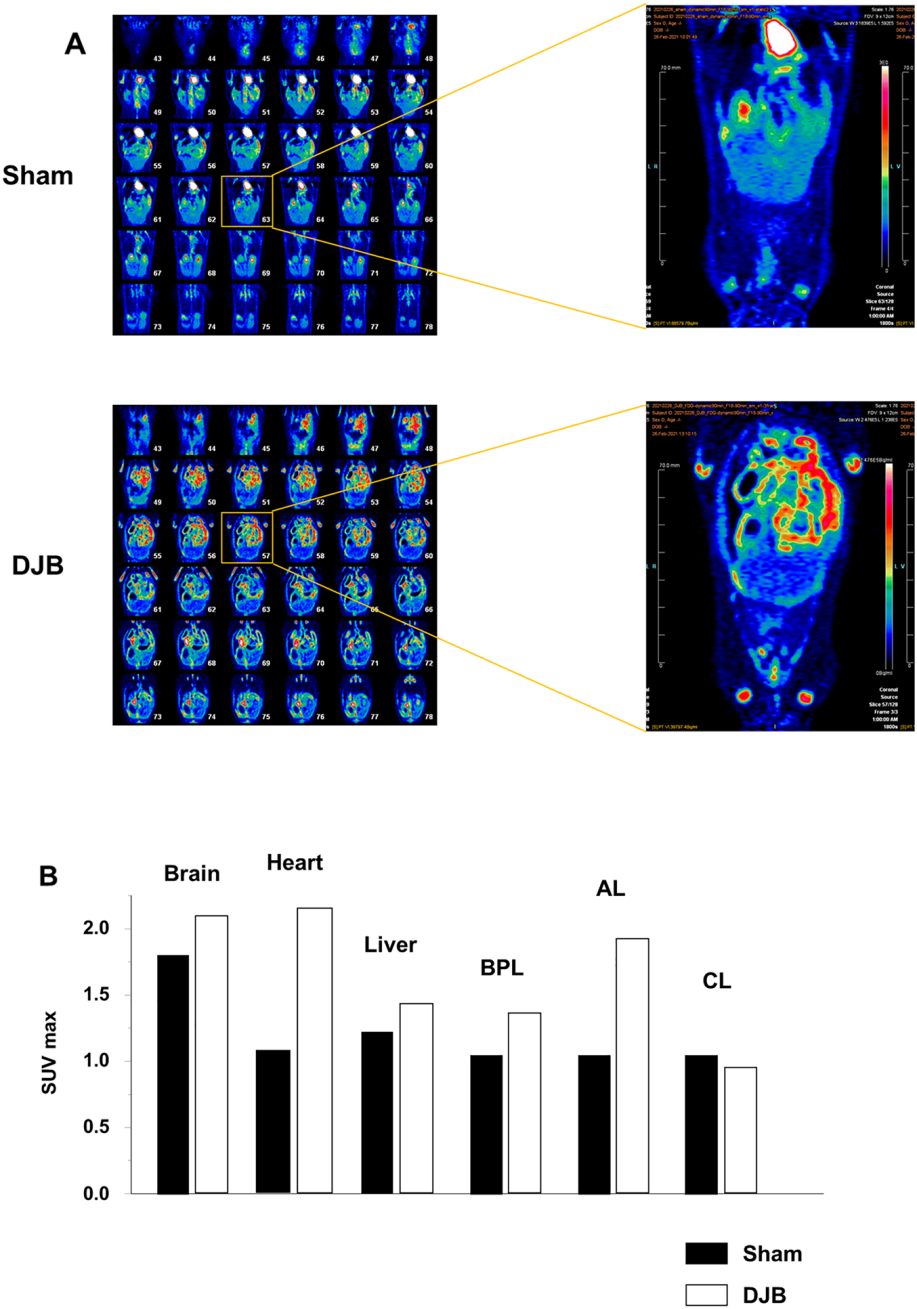
Positron emission tomography-computed tomography (PET-CT) showed that glucose uptake by the proximal jejunum was higher very soon after duodenal-jejunal bypass (DJB). (**A**) Representative images obtained from whole-body PET/CT scanning of a sham-operated and a DJB-operated rat. [18F]-fluorodeoxyglucose uptake was high in the proximal jejunum of DJB-operated rats. (**B**) Comparison of the maximum standardized uptake values (SUVmax) of major organs after surgery between the sham- and DJB-operated rats.

To determine whether changes in glucose uptake and metabolic activity are associated with the changes in glucose transporter expression in the proximal jejunum of rats that underwent DJB, [18F]-FDG PET-CT was performed in both groups. Whole-body [18F]-FDG PET scanning showed a marked increase in FDG uptake in the proximal small intestine in DJB-operated rats versus sham-operated rats (Fig. 3A). The SUVmax values associated with [18F]-FDG uptake in the brain, heart, liver, and each limb of the small intestine were measured (Fig. 3B), which showed that sham-operated rats had much higher glucose uptake by the brain than by other organs, whereas the DJB-operated rats showed similar glucose uptake by the heart and AL to that by the brain. These findings imply that changes in glucose transporter expression, and especially an increase in GLUT1 expression, is followed by intestinal remodeling, secondary to increases in intestinal glucose uptake and utilization, during the very early postoperative period following DJB surgery.

## Discussion

In the present study, we determined the effect of DJB during very early postoperative period in rats with T2DM. The fasting blood glucose concentrations on POD1 were significantly lower in the DJB group than in the sham group. OGTT showed that DJB rapidly improved glucose tolerance after surgery, without improving insulin resistance or secretion. The expression of SGLT1 and GLUT1 was upregulated and GLUT2 expression was downregulated in the AL of the DJB group *versus* the sham group. [18F]-FDG PET-CT showed that the FDG uptake had been increased in the AL of the DJB group to a level similar to that of the brain. Most importantly, these changes that may lead to improvement of T2DM occurred very early post operative period, 1 day after surgery.

Metabolic surgery has been reported to reduce blood glucose concentration more effectively than intensive medical therapy and lifestyle interventions^21–23^. Furthermore, over 80% of patients who undergo metabolic surgery were reported to maintain good long-term control of glycemia without the need for significant medication^24,25^. Interestingly, the effect of metabolic surgery on hyperglycemia appears within a few days of surgery, despite the absence of weight loss at that time^26,27^. However, the detailed mechanisms underlying this rapid improvement in T2DM have not been determined^28–30^. In the present study, we have confirmed that DJB ameliorates hyperglycemia in the very early postoperative period (on 1POD) in GK rats with T2DM, but with no difference in insulin concentration. Several studies have shown that DJB improves glucose tolerance, without increasing insulin secretion, 2 weeks after surgery in GK rats^8,31^. Breen et al. also reported that DJB rapidly improves hyperglycemia in rats with streptozotocin-induced T1DM, which are insulin deficient, and Sprague Dawley rats on 2POD, and revealed that these effects were achieved not through changes in insulin concentration, body mass, or food intake, but by a reduction in endogenous glucose concentration, caused by diverting nutrient flow directly into the jejunum^32^. These findings suggest that the effect of DJB on blood glucose concentration during the very early postoperative period may be the result of altered jejunal nutrient sensing and changes in glucose flux, independently of weight loss or improvements in insulin resistance or secretion. Therefore, we focused on the postoperative remodeling of the intestine.

**Figure 4:**
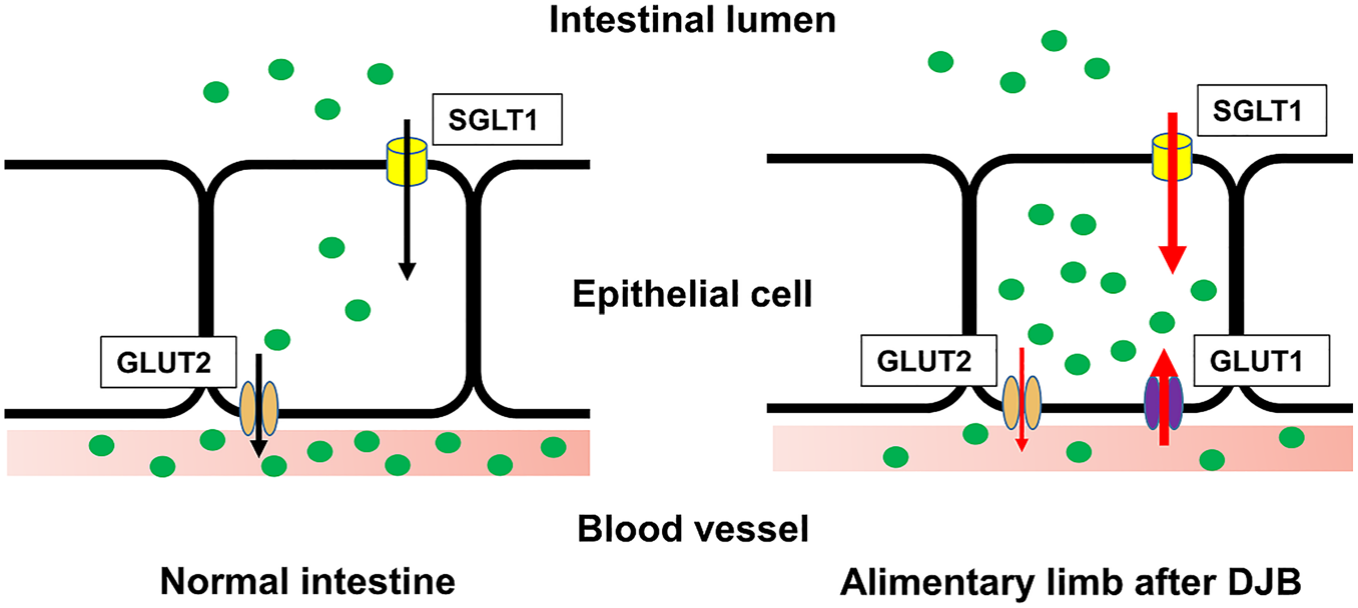
Schematic view of the intestinal responses associated with the improvement in glucose tolerance during the very early postoperative period following duodenal-jejunal bypass (DJB). In response to DJB, the glucose transporter expression in the epithelial cells of the alimentary limb increases and glucose pools in the epithelial cells of the intestine. This accumulation of glucose in intestinal epithelial cells may contribute to the improvement in glucose tolerance that occurs rapidly after DJB.

Several studies have demonstrated that RYGB induces hyperplasia of the intestinal tract^14,33,34^, and RYGB, like DJB, is a form of metabolic surgery that involves the removal of the duodenum and a redirection of nutrient flow to the jejunum. Recently, it has been revealed that the post-surgical remodeling of the intestinal tract involve not only morphological changes, but also metabolic changes^12,13^. Saeidi et al. showed that the upregulation of GLUT1 expression in the AL 2 months after RYGB facilitates intestinal glucose uptake and that the improvement in postoperative blood glucose concentration positively correlates with intestinal glucose uptake^13^. Cavin et al. reported that the increase in the intestinal glucose consumption of AL 2 weeks after RYGB is the result of higher expression of GLUT1 and SGLT1 and is associated with better glucose tolerance. In contrast, SG, a metabolic surgery that does not involve the diversion of nutrient flow, does not cause intestinal remodeling or an increase in glucose uptake by the intestine^12^. In the present study, we have shown changes in glucose transporter expression in association with glucose accumulation in the intestine only 1 day following DJB (Fig. 4), implying that glucose transporter expression, along with intestinal remodeling, start in the very early postoperative period.

It has been reported that the increase in glucose uptake that occurs in the small intestine is principally mediated via GLUT1^12,13,35^, and in the present study, GLUT1 expression was higher in the BPL and AL, which are in the proximal jejunum, in the DJB group. To investigate the contribution of the greater intestinal glucose uptake to whole-body glucose consumption, we performed PET-CT using [18F]-FDG. This showed that the proximal jejunum of the DJB group transported more [18F]-FDG than that of the sham group during the early postoperative period. In particular, the AL of the DJB group showed much higher [18F]-FDG uptake, becoming one of the organs with the highest glucose consumption. Several studies have shown that [18F]-FDG uptake is upregulated in AL, because of higher GLUT1 expression, after RYGB^12,13^, which is consistent with the present findings. GLUT1 is usually abundantly expressed in the fetal intestine, but gradually disappears, until it is barely detectable in the adult intestine^20^. GLUT1 is considered to play a role in early intestinal growth, and it has been reported that intestinal GLUT1 expression is high in patients with short bowel syndrome^36^. Intestinal shortening or bypass of the normal dietary route may cause the upregulation of GLUT1 after RYGB or DJB, given that this does not occur following SG. While the crucial role of GLUT1 expression changes in improvement of T2DM after metabolic surgery is becoming increasingly clear, the role of SGLT1 expression changes remains controversial. Cavin et al. demonstrated that the upregulation of apical SGLT1 and basolateral GLUT1 expressions increase glucose uptake both from intraluminal glucose and blood glucose, and accumulate glucose in mucosa to satisfy their energy requirements for remodeling^12^. In the present study, changes in glucose transporter expression in AL suggested that much amount of absorbed glucose could remain within epithelial cells, supporting the previous study that increased glucose uptake into the intestinal mucosa is important for intestinal remodeling. On the contrary, Jiang et al. reported that the SGLT1 expression in AL was downregulated 6 weeks after DJB surgery and suggested decreased intestinal glucose uptake could induce intestinal remodeling^37^. These conflicting findings suggest that the mechanism leading to improvement of T2DM may be different between early and later postoperative periods.

The vagus nerve is reported to be activated by the rapid exposure of the small intestine to undigested food following surgeries that alters nutrient flow, such as RYGB and DJB^38,39^. The vagus nerve has been reported to permit bidirectional signaling between the gut and the brain, and therefore to be involved in the rapid regulation of systemic energy control, including that of glucose metabolism^40–42^. Borgmann et al. demonstrated that the activation of vagal afferents causes a rapid reduction in blood glucose concentration within 1 h through the upregulation of glucose uptake in the skeletal muscle of mice, without increasing their insulin concentrations, which is consistent with the present findings^40^. Several previous studies have shown that vagal afferents are involved in the regulation of SGLT1 expression^43,44^. Tavakkolizadeh et al. revealed that vagal afferents regulate GLUT2 expression in the small intestine of rats^45^, and Yang et al. reported that the activation of vagal afferents causes the upregulation of GLUT1 expression in the myocardium of diabetic mice^46^. These studies suggest that the activation of vagal afferents by the rapid exposure of the small intestine to nutrients following metabolic surgery may cause the upregulation of glucose transporter expression and increase glucose uptake in the small intestine. In addition, previous study has shown that RYGB significantly increases serum bile acid concentrations and dramatically alters the proportions of each bile acid component^47^. These changes of bile acid were also observed after DJB^48^, and bile acids have been suggested as a factor that affect glucose transporters and activity^49,50^. Further investigations are needed to understand the underlying mechanisms involved in changes of glucose transporter after metabolic surgery.

This study had several limitations. First, we measured mRNA levels of glucose transporters after surgery. Measuring protein levels would further strengthened this study, because mRNA turnover may differ after surgery without affecting total protein levels. Second, we measured plasma insulin concentrations during OGTT. These may not accurately reflect pancreatic function because insulin clearance changes after metabolic surgery.

In conclusion, the present study demonstrated the effects of DJB on T2DM rats in the very early postoperative period. DJB rapidly improves glucose tolerance on 1POD, without improving insulin secretion or resistance. The expression of glucose transporters in the small intestine were significantly affected by DJB, and [18F]-FDG PET-CT revealed that FDG uptake is upregulated in the proximal jejunum after DJB. These changes may contribute to the reduction in hyperglycemia by increasing glucose uptake in the small intestine (Fig. 4). Therefore, we have shown that the rapid improvement in T2DM induced by DJB may be the result of changes in glucose transporter expression, resulting in greater glucose uptake by the small intestine.

## Methods

### Animals

Male 15–18-week-old Goto-Kakizaki (GK) rats were purchased from CREA Japan, Inc. (Tokyo, Japan). They had free access to food and water and were kept under conditions of controlled temperature and humidity, and a 12-h light/dark cycle. The study was approved by the Department of Animal Research Resources, Institute of Health Biosciences, Tokushima University, performed according to the relevant guidelines and regulations, and complied with the ARRIVE guidelines.

### DJB and sham surgery

**Figure 5:**
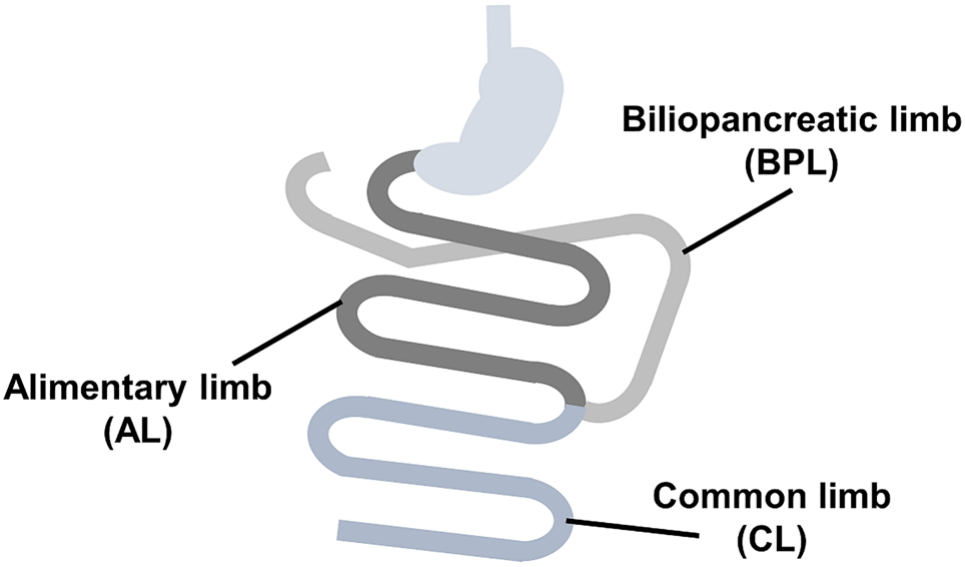
Schematic of the duodenal-jejunal bypass. The alimentary limb (AL) is the part between the duodenal-jejunal anastomosis and the jejunal–jejunal anastomosis, the biliopancreatic limb (BPL) is the part between the duodenal stump and the jejunal–jejunal anastomosis, and the common limb (CL) is the part between the jejunal–jejunal anastomosis and the terminal ileum.

A total of eight rats were randomly allocated to two groups: a sham group and a DJB group. They were fasted overnight and underwent surgery under anesthesia with 2–3% isoflurane in air/oxygen. In the sham-operated group, a midline laparotomy was performed, then the skin incision was closed. In the DJB group, the procedure was performed as described by Rubino et al.^51^. Briefly, the duodenum was separated just distal to the pylorus, then the distal duodenum was closed. The jejunum was cut 10 cm from the ligament of Treitz and the distal jejunum was connected to the proximal duodenum to create an alimentary limb (AL). A biliopancreatic limb (BPL) was created by joining the proximal jejunum to the remaining jejunum 15 cm distal to the duodenojejunostomy. The common limb (CL) was defined as the intestine distal to the jejunojejunostomy (Fig. 5). The rats were given free access to food and water from just after surgery.

### Oral glucose tolerance testing (OGTT)

OGTT was performed on 1POD after overnight fasting. A 50% glucose solution (2 g/kg) was administered to the rats using a polyethylene gastric tube, then blood samples were collected from a tail vein 0, 15, 30, 60, and 120 min later into Eppendorf tubes containing aprotinin and EDTA. Blood glucose concentrations were measured at the same time points using a Medi Safe GR-102 Mini glucometer (Terumo, Tokyo, Japan), and plasma was prepared by centrifugation (2000×*g*, 15 min) and stored at − 80 °C until use.

### ELISA

The plasma concentrations of insulin were measured using an AKRIN-010T Rat Insulin ELISA kit (Shibayagi, Gunma, Japan), according to the manufacturer’s protocol. A microplate reader (SpectraMax i3; Molecular Devices, LLC, San Jose, CA, USA) was used to measure the absorbances at 450 nm. The degree of insulin resistance was assessed using the homeostatic model assessment of insulin resistance (HOMA-IR), as: HOMA-IR = [fasting glucose (mg/dl) × fasting insulin (μIU/ml)]/405.

### Reverse transcription-quantitative polymerase chain reaction (RT-qPCR)

RT-qPCR was performed as previously described^52^. Briefly, an RNeasy Mini Kit (Qiagen, Hilden, Germany) was used to extract RNA, according to the manufacturer’s instructions. A reverse transcription kit (Applied Biosystems, Thermo Fisher Scientific Inc., Waltham, MA, USA) was used to synthesize cDNA. The TaqMan assays used were: *Sglt1* (Rn00564718_m1), GLUT1 (Rn01417099_m1), and GLUT2 (Rn00563565_m1). GAPDH (4326317E) was used as the reference gene. The primer sets were obtained from Thermo Fisher Scientific, Inc. The results are presented as fold differences in relative mRNA expression *versus* the sham group.

### Immunohistochemistry

Samples were fixed in 10% formalin and embedded in paraffin, then paraffin sections were prepared at 4-μm thickness, deparaffinized using xylene, and dehydrated using a series of graded ethanol solutions. Endogenous peroxidase activity was blocked by incubation in 0.3% hydrogen peroxidase and methanol for 20 min. After washing with phosphate-buffered saline (PBS; Fisher Scientific, Pittsburgh, PA, USA), antigen retrieval was performed in citrate buffer (pH 6.0) in a microwave oven for 15 min. After cooling to room temperature, the sections were incubated with primary rabbit monoclonal antibodies targeting SGLT1 (GTX33495, 1:100; GeneTex, Irvine, CA, USA), GLUT1 (ab115730, 1:500; Abcam, Cambridge, UK), or GLUT2 (20436-1-AP, 1:500; Proteintech, Chicago, IL, USA) at 4 °C overnight. The sections were then incubated with secondary antibody (REAL EnVision/HRP detection system; Dako, Tokyo, Japan) for 1 h. After three washes with PBS, 3,3′-diaminobenzidine tetrahydrochloride (DAB) was added to identify the proteins and nuclei were counterstained using Mayer’s hematoxylin solution.

### Positron emission tomography-computed tomography (PET-CT)

[18F]-fluorodeoxyglucose ([18F]-FDG) uptake was measured in DJB- or sham-operated diabetic rats (n = 1, respectively). Twenty-four hours after surgery, and after fasting overnight with access to water only, the rats were subjected to [18F]-FDG PET-CT using a Siemens Inveon Small-animal PET scanner (Siemens Healthcare, Knoxville, TN, USA). The PET-CT procedure and analysis of the maximum standardized uptake value (SUVmax) were performed as described as previously described^53^. Briefly, the rats were anesthetized using 1.5–2.0% isoflurane and injected via a tail-vein catheter with 20 MBq/0.1–0.2 ml 18F-FDG. PET data were acquired for 20 min following a delay of 40 min to permit FDG uptake. SUVmax was calculated using the maximum voxel value (Bq/ml) for each organ.

### Statistical analysis

Statistical comparisons were made using unpaired Student’s *t*-tests. Data are expressed as mean ± SD. *P* < 0.05 was considered to represent statistical significance. Statistical analyses were performed using JMP software (version 13; SAS Campus Drive, Cary, NC, USA).

## Data Availability

The datasets generated and/or analyzed during the current study are available from the corresponding author on reasonable request.

## References

[1] DeFronzo RA (2004). Pathogenesis of type 2 diabetes mellitus. Med. Clin. N. Am..

[2] Tripathi BK, Srivastava AK (2006). Diabetes mellitus: Complications and therapeutics. Med Sci Monit..

[3] Wild S, Roglic G, Green A, Sicree R, King H (2004). Global prevalence of diabetes: Estimates for the year 2000 and projections for 2030. Diabetes Care.

[4] Saeedi P (2019). Global and regional diabetes prevalence estimates for 2019 and projections for 2030 and 2045: Results from the International Diabetes Federation Diabetes Atlas, 9th edition. Diabetes Res. Clin. Pract..

[5] Cefalu WT, Rubino F, Cummings DE (2016). Metabolic surgery for type 2 diabetes: Changing the landscape of diabetes Care. Diabetes Care.

[6] Rubino F, Cummings DE (2012). Surgery: The coming of age of metabolic surgery. Nat. Rev. Endocrinol..

[7] Rubino F, Marescaux J (2004). Effect of duodenal-jejunal exclusion in a non-obese animal model of type 2 diabetes: A new perspective for an old disease. Ann. Surg..

[8] Guan W (2020). Duodenal-jejunal exclusion surgery improves type 2 diabetes in a rat model through regulation of early glucose metabolism. Can. J. Diabetes..

[9] Dolo PR, Yao L, Li C, Zhu X, Shi L, Widjaja J (2018). Preserving duodenal-jejunal (foregut) transit does not impair glucose tolerance and diabetes remission following gastric bypass in type 2 diabetes Sprague Dawley rat model. Obes. Surg..

[10] Kashihara H (2014). Duodenal-jejunal bypass improves insulin resistance by enhanced glucagon-like peptide-1 secretion through increase of bile acids. Hepatogastroenterology..

[11] Seki Y, Kasama K, Umezawa A, Kurokawa Y (2016). Laparoscopic sleeve gastrectomy with duodenojejunal bypass for type 2 diabetes mellitus. Obes. Surg..

[12] Cavin J-B (2016). Differences in alimentary glucose absorption and intestinal disposal of blood glucose following Roux-en-Y gastric bypass vs sleeve gastrectomy. Gastroenterology.

[13] Saeidi N (2013). Reprogramming of intestinal glucose metabolism and glycemic control in rats after gastric bypass. Science.

[14] Mumphrey MB, Hao Z, Townsend RL, Patterson LM, Berthoud H-R (2015). Sleeve gastrectomy does not cause hypertrophy and reprogramming of intestinal glucose metabolism in rats. Obes. Surg..

[15] Hansen CF (2013). Hypertrophy dependent doubling of L-cells in Roux-en-Y gastric bypass operated rats. PLoS ONE.

[16] Kellett GL (2001). The facilitated component of intestinal glucose absorption. J. Physiol..

[17] Kellett GL, Brot-Laroche E (2005). Apical GLUT2: A major pathway of intestinal sugar absorption. Diabetes.

[18] Koepsell H (2020). Glucose transporters in the small intestine in health and disease. Pflugers Arch..

[19] Wright EM, Loo DDF, Hirayama BA (2011). Biology of human sodium glucose transporters. Physiol. Rev..

[20] Thorens B (1996). Glucose transporters in the regulation of intestinal, renal, and liver glucose fluxes. Am. J. Physiol..

[21] Courcoulas AP, Belle SH, Neiberg RH (2015). Three-year outcomes of bariatric surgery vs lifestyle intervention for type 2 diabetes mellitus treatment: A randomized clinical trial. JAMA Surg..

[22] Cummings DE (2016). Gastric bypass surgery vs intensive lifestyle and medical intervention for type 2 diabetes: the CROSSROADS randomised controlled trial. Diabetologia.

[23] Fruhbeck G (2015). Bariatric and metabolic surgery: A shift in eligibility and success criteria. Nat. Rev. Endocrinol..

[24] Mingrone G (2015). Bariatric-metabolic surgery versus conventional medical treatment in obese patients with type 2 diabetes: 5 year follow-up of an open-label, single-centre, randomised controlled trial. Lancet.

[25] Schauer PR (2017). Bariatric surgery versus intensive medical therapy for diabetes—5-year outcomes. N. Engl. J. Med..

[26] Schauer PR (2003). Effect of laparoscopic Roux-en-Y gastric bypass on type 2 diabetes mellitus. Ann. Surg..

[27] Fried M, Ribaric G, Buchwald JN, Svacina S, Dolezalova K, Scopinaro N (2010). Metabolic surgery for the treatment of type 2 diabetes in patients with BMI < 35 kg/m^2^: An integrative review of early studies. Obes. Surg..

[28] Laferrère B (2011). Do we really know why diabetes remits after gastric bypass surgery?. Endocrine.

[29] Cummings DE (2012). Metabolic surgery for type 2 diabetes. Nat. Med..

[30] Bradley D (2012). Gastric bypass and banding equally improve insulin sensitivity and β cell function. J. Clin. Investig..

[31] Han H (2015). Expedited biliopancreatic juice flow to the distal gut benefits the diabetes control after duodenal-jejunal bypass. Obes. Surg..

[32] Breen DM, Rasmussen BA, Kokorovic A, Wang R, Cheung GWC, Lam TKT (2012). Jejunal nutrient sensing is required for duodenal-jejunal bypass surgery to rapidly lower glucose concentrations in uncontrolled diabetes. Nat. Med..

[33] Taqi E (2010). The influence of nutrients, biliary-pancreatic secretions, and systemic trophic hormones on intestinal adaptation in a Roux-en-Y bypass model. J. Pediatr. Surg..

[34] Bueter M (2010). Gastric bypass increases energy expenditure in rats. Gastroenterology.

[35] Kwon IG (2021). Serum glucose excretion after Roux-en-Y gastric bypass: A potential target for diabetes treatment. Gut.

[36] Sanaksenaho G (2020). Parenteral nutrition-dependent children with short-bowel syndrome lack duodenal-adaptive hyperplasia but show molecular signs of altered mucosal function. JPEN J. Parenter. Enteral. Nutr..

[37] Jiang B (2022). Role of proximal intestinal glucose sensing and metabolism in the blood glucose control in type 2 diabetic rats after duodenal jejunal bypass surgery. Obes. Surg..

[38] Tack J, Arts J, Caenepeel P, Wulf DD, Bisschops R (2009). Pathophysiology, diagnosis and management of postoperative dumping syndrome. Nat. Rev. Gastroenterol. Hepatol..

[39] Tack J, Deloose E (2014). Complications of bariatric surgery: Dumping syndrome, reflux and vitamin deficiencies. Best Pract. Res. Clin. Gastroenterol..

[40] Borgmann D (2021). Gut-brain communication by distinct sensory neurons differently controls feeding and glucose metabolism. Cell Metab..

[41] Wang PY (2008). Upper intestinal lipids trigger a gut–brain–liver axis to regulate glucose production. Nature.

[42] Soty M, Gautier-Stein A, Rajas F, Mithieux G (2017). Gut–brain glucose signaling in energy homeostasis. Cell Metab..

[43] Stearns AT, Balakrishnan A, Rhoads DB (2010). Rapid upregulation of sodium-glucose transporter SGLT1 in response to intestinal sweet taste stimulation. Ann. Surg..

[44] Stearns AT, Balakrishnan A, Rhoads DB, Tavakkolizadeh A (2010). Rapid upregulation of sodium-glucose transporter SGLT1 in response to intestinal sweet taste stimulation. Ann. Surg..

[45] Stearns AT, Balakrishnan A, Rounds J, Rhoads DB, Ashley SW, Tavakkolizadeh A (2008). Capsaicin-sensitive vagal afferents modulate posttranscriptional regulation of the rat Na^+^/glucose cotransporter SGLT1. Am. J. Physiol. Gastrointest. Liver Physiol..

[46] Yang Y (2019). Pyridostigmine regulates glucose metabolism and mitochondrial homeostasis to reduce myocardial vulnerability to injury in diabetic mice. Am. J. Physiol. Endocrinol. Metab..

[47] Simonen M (2012). Conjugated bile acids associate with altered rates of glucose and lipid oxidation after Roux-en-Y gastric bypass. Obes. Surg..

[48] Chai J (2015). Mechanism of bile acid-regulated glucose and lipid metabolism in duodenal-jejunal bypass. Int. J. Clin. Exp. Pathol..

[49] Chadt A, Al-Hasani H (2020). Glucose transporters in adipose tissue, liver, and skeletal muscle in metabolic health and disease. Pflugers Arch..

[50] Ding L, Yang L, Wang Z, Huang W (2015). Bile acid nuclear receptor FXR and digestive system diseases. Acta Pharm. Sin. B..

[51] Rubino F (2006). The mechanism of diabetes control after gastrointestinal bypass surgery reveals a role of the proximal small intestine in the pathophysiology of type 2 diabetes. Ann. Surg..

[52] Okikawa S (2022). Inhibition of the VEGF signaling pathway attenuates tumor-associated macrophage activity in liver cancer. Oncol. Rep..

[53] Otani T (2019). Non-invasive monitoring of cisplatin and erlotinib efficacy against lung cancer in orthotopic SCID mouse models by small animal FDG-PET/CT and CT. Oncol. Rep..

